# Traumatic hepatic hernia formation following abdominal trauma: A case report

**DOI:** 10.1097/MD.0000000000045257

**Published:** 2025-10-24

**Authors:** Dezheng Liu, Shukuan Yang, Zijian Zhao, Yangyang Cui, Xuekai Zhao, Fan Zhang, Qinghai Guan, Lei Zhou

**Affiliations:** a Department of Biliopancreatic Surgery, Binzhou Medical University Hospital, Binzhou, China; b Department of Hepatobiliary Surgery, Binzhou People’s Hospital, Binzhou, China.

**Keywords:** abdominal trauma, computed tomography, hepatic hernia, traumatic abdominal wall hernia

## Abstract

**Rationale:**

Abdominal trauma and hernia formation represent relatively common clinical entities. However, traumatic abdominal wall hernia resulting from abdominal injury, specifically involving hepatic protrusion through the abdominal wall, is sparsely documented in the literature. The rarity of such cases, often due to high-impact forces and unique shear mechanisms, makes them noteworthy for surgical documentation and review. Following diagnosis, abdominal wall hernias typically necessitate surgical intervention to reduce the herniated contents and reconstruct the compromised wall, often with mesh reinforcement. Left untreated, these hernias pose a risk of potentially serious complications such as incarceration, strangulation, or bowel ischemia. Therefore, prompt recognition and timely surgical repair are critical to prevent life-threatening sequelae and ensure optimal patient outcomes.

**Patient concerns:**

A 57-year-old female patient presented 9 hours prior to admission following a bicycle collision, sustaining right flank trauma. Physical examination revealed a 6 × 7 cm subcutaneous ecchymosis at the impact site, and the patient experienced severe pain.

**Diagnoses:**

Preoperative computed tomography and abdominal ultrasonography revealed focal protrusion of the right hepatic lobe through the abdominal wall. Intraoperative laparoscopic examination confirmed the diagnosis of hepatic herniation. The hernia was characterized by protrusion of liver segment V into the anterior abdominal wall, with dense adhesions to the abdominal wall.

**Interventions:**

The patient underwent laparoscopic surgery, during which laparoscopic exploration confirmed the diagnosis of hepatic herniation. Subsequently, reduction of the herniated liver segment and surgical repair of the resulting abdominal wall defect were performed.

**Outcomes:**

Postoperatively, the patient experienced marked reduction in abdominal pain. Follow-up computed tomography demonstrated restoration of normal hepatic anatomical architecture. Liver function tests and routine biochemistry panels revealed no significant abnormalities.

**Lessons:**

Traumatic hepatic herniation represents an uncommon clinical entity, frequently leading to inadequate vigilance among clinicians regarding rare complications during routine management of abdominal trauma. Consequently, early and comprehensive utilization of imaging modalities, supplemented by minimally invasive diagnostic or therapeutic techniques when indicated, is essential to achieve precise diagnosis and management within a precision medicine framework.

## 1. Introduction

Blunt or penetrating abdominal trauma, resulting from diverse etiologies, represents a frequent clinical entity. Common complications include visceral injuries (such as rupture of solid organs [e.g., liver, spleen, kidneys] causing intra-abdominal hemorrhage, or perforation of hollow viscera [e.g., stomach, intestines] leading to chemical/bacterial peritonitis). These typically present with abdominal pain, pallor, hypotension, or even shock. Such manifestations are routinely incorporated into surgical differential diagnoses and management protocols.

However, hepatic herniation through a diaphragmatic defect with protrusion into the anterolateral abdominal wall following abdominal trauma remains exceedingly rare. Herein, we present a case of traumatic hepatic hernia and discuss its clinical characteristics, diagnostic challenges, and therapeutic strategies through a comprehensive literature review.

## 2. Case presentation

### 2.1. History

A 57-year-old female patient presented to our institution with a history of abdominal trauma. She reported a bicycle fall approximately 9 hours prior, resulting in blunt abdominal trauma and severe pain. Physical examination revealed a 6 × 7 cm subcutaneous ecchymosis in the right upper quadrant. Vital signs were stable, and no other significant abnormalities were identified. The patient’s medical history was unremarkable.

### 2.2. Examination findings

Routine laboratory investigations revealed aspartate aminotransferase at 94.30 U/L and alanine aminotransferase at 85.00 U/L, accompanied by reduced red blood cell count (3.6 × 10^12^ L) and hemoglobin level (107 g/L). These findings suggested mild hepatic impairment and possible minor blood loss.

Serological testing for HIV, HBV, and HCV antibodies, as well as coagulation parameters, were within normal limits.

**Figure 1.**
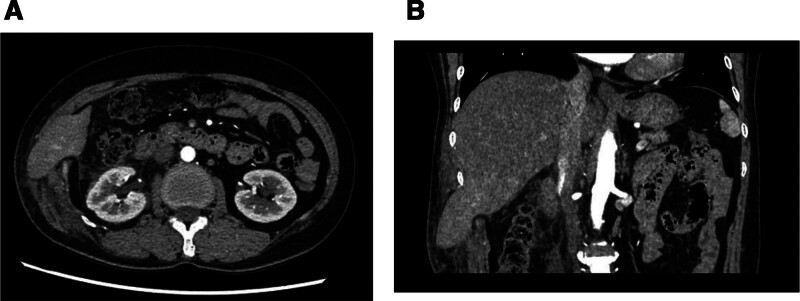
(A and B) CT revealed herniation of the anterolateral segment of the right hepatic lobe through a diaphragmatic defect, accompanied by significant soft tissue edema and fat stranding within the adjacent right abdominal wall. CT = computed tomography.

Contrast-enhanced computed tomography (CT) of the upper abdomen demonstrated focal herniation of the right hepatic lobe with concomitant soft tissue edema/inflammatory changes in the right abdominal wall (Fig. 1A and B).

### 2.3. Treatment course

The patient was provisionally diagnosed with hepatic herniation requiring surgical intervention. However, due to significant abdominal wall edema and fluid exudation, immediate surgery was deemed contraindicated following multidisciplinary assessment. Conservative management was initiated, comprising fluid resuscitation and hepatoprotective therapy. The patient was discharged for outpatient observation with instructions for restricted activity for 1 month.

**Figure 2.**
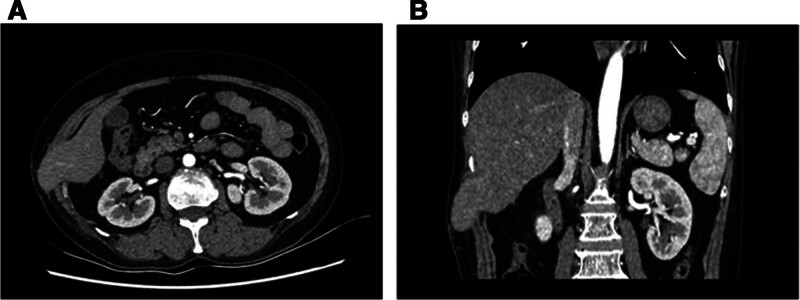
(A and B) One month later, significant resolution of abdominal wall edema and exudation was observed.

One month later, repeat contrast-enhanced abdominal CT demonstrated persistent herniation of the right hepatic lobe but showed marked resolution of abdominal wall edema and inflammatory changes (Fig. 2A and B).

**Figure 3.**
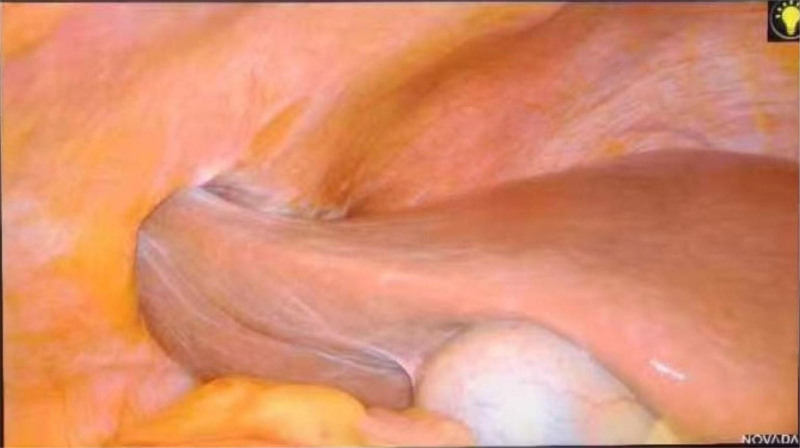
Laparoscopic visualization confirmed incarceration of the anterolateral segment V hepatic parenchyma within the abdominal wall defect, demonstrating dense fibrotic adhesions to the surrounding musculofascial layers with associated marked inflammatory reaction in adjacent tissues.

Upon entering the peritoneal cavity, incarceration of segment V hepatic parenchyma within the abdominal wall defect was observed, with dense adhesions to the surrounding tissues and associated inflammatory reaction (Fig. 3). Systematic exploration of the remaining hepatic segments, gastrointestinal tract, and peritoneal cavity revealed no macroscopic abnormalities.

**Figure 4.**
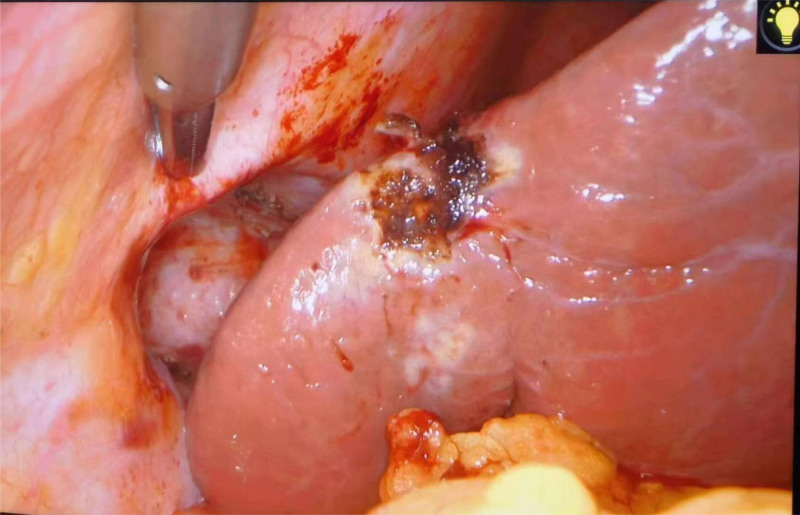
The liver was successfully restored to its native anatomical position.

Following definitive diagnosis, adhesions between the hepatic parenchyma and abdominal wall were meticulously dissected using an electrocautery hook. Hemostasis was meticulously achieved at the dissection site. The incarcerated hepatic tissue was then reduced, and the hernial sac was ligated. Laparoscopic visualization confirmed restoration of the liver to its normal anatomical position (Fig. 4).

**Figure 5.**
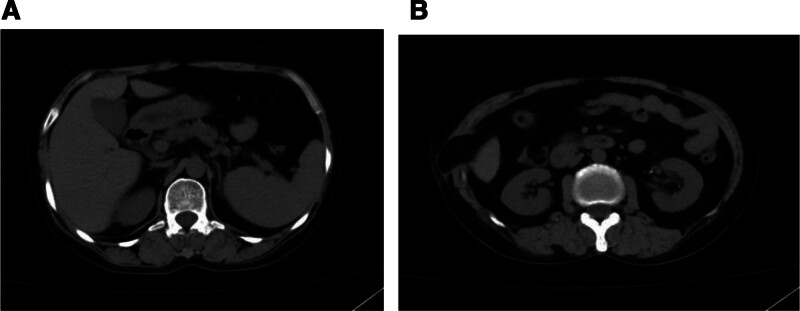
(A and B) Postoperative CT confirmed complete reduction of the hepatic parenchyma into the anterosuperior subphrenic space with restoration of normal anatomical relationships. CT = computed tomography.

Postoperative contrast-enhanced abdominal CT demonstrated complete reduction of the herniated liver and restoration of intraperitoneal anatomy (Fig. 5A and B).

### 2.4. Follow-up

Postoperatively, the patient experienced complete resolution of abdominal pain and progressive regression of subcutaneous ecchymosis. Follow-up laboratory investigations, including complete blood count, comprehensive metabolic panel, and liver function tests, demonstrated normalization of all previously deranged parameters, with hepatic enzyme levels returning to physiologic ranges.

## 3. Discussion

### 3.1. Our case in the context of existing literature

Abdominal trauma typically prompts clinical surgeons to evaluate for associated visceral injuries, with renal, splenic, and hepatic involvement demonstrating sequentially decreasing incidence rates. These injuries frequently manifest with acute abdominal pain, localized erythema/edema, and hypotension. Injuries to the pancreas, bladder, or intestinal tract are comparatively uncommon.^[1]^ Concurrently, discussions of hernia pathology predominantly focus on inguinal, femoral, or umbilical variants,^[2]^ often overlooking trauma-induced hernias as a distinct entity. Herein, we present a clinically significant case of traumatic hepatic herniation secondary to abdominal trauma, supplemented by a systematic review of prior literature, to establish a feasible therapeutic approach for this condition.

### 3.2. Clinical presentation of traumatic hepatic hernia

Guzman-Nuño et al suggested in their studies^[3,4]^ that hepatic herniation in individuals ≤ 18 years of age is closely associated with congenital diaphragmatic hernia or congenital abdominal wall defects. In contrast, among individuals > 18 years of age, the formation of hepatic hernia is frequently associated with postoperative incisional hernias following upper abdominal surgery and abdominal trauma.^[5,6]^ Hepatic hernias resulting from abdominal trauma are typically accompanied by abdominal pain and hematoma or ecchymosis in the subcutaneous tissue. Furthermore, compared to visceral injuries caused by trauma, hepatic hernia lacks distinguishing clinical characteristics.

### 3.3. The central role of computed tomography

Computed tomography plays a crucial role in the diagnosis of hepatic herniation. In the previously cited cases, this modality was consistently employed. CT enables rapid localization of the herniated liver segment, delineates its size, and facilitates assessment of inflammatory exudation at the lesion site, thereby aiding in determining the optimal timing for surgical intervention.

In the present case, the initial admission CT scan revealed herniation of liver segment V protruding towards the abdominal wall with dense adhesions to it. However, concomitant findings of significant abdominal wall edema and substantial fluid exudation prompted the decision to defer immediate surgical repair.

Upon the patient’s second admission, a follow-up CT scan demonstrated marked resolution of abdominal wall edema and near-complete absorption of the exudative fluid. Surgical intervention was subsequently performed at this stage. A postoperative CT scan confirmed successful reduction of the liver from the hernial sac and restoration of its normal anatomical position.

In summary, CT imaging represents the diagnostic modality of choice for hepatic hernia, applicable to both preoperative evaluation and postoperative assessment.

Notably, isolated reports exist describing the misdiagnosis of hepatic hernia as pulmonary neoplasms or pulmonary cysts on CT imaging.^[7–9]^ These instances highlight the imperative for integrating ancillary diagnostic findings and caution against relying solely on CT findings for definitive diagnosis.

### 3.4. Surgical intervention as definitive treatment

Following diagnosis, hepatic herniation typically necessitates surgical intervention to achieve anatomical reduction. Reports indicate that conservative management may lead to adverse outcomes; 1 documented case^[10]^ developed strangulation necrosis of the herniated liver segment following nonoperative treatment, resulting in intra-abdominal infection. This complication was ultimately controlled only after percutaneous drainage and subsequent surgical intervention. Notably, our review of published hepatic hernia case reports revealed that all patients achieved definitive cure solely through surgical repair. Consequently, we propose that surgical intervention represents the only definitive curative approach for this condition. Concomitantly, a comprehensive preoperative assessment of the patient’s overall condition is imperative. This evaluation must encompass parameters such as liver function tests, coagulation profile, and complete blood count to ensure values fall within normal ranges. Additionally, evaluation for potential concomitant visceral injuries sustained during the abdominal trauma and assessment of abdominal wall inflammatory changes and edema are essential. Should any of these parameters demonstrate significant abnormalities, surgical intervention should be judiciously postponed. Surgery may then be undertaken once the patient’s condition has been optimized and the aforementioned abnormalities have resolved or stabilized.

### 3.5. Available repair techniques and outcomes

Based on our review of the relevant literature, the currently available repair techniques include autologous tissue repair using prosthetic mesh reinforced with peririb cable straps,^[11]^ open transthoracic patch repair,^[12]^ and tension-free laparoscopic repair with absorbable mesh,^[13]^ among other approaches. In the present case report, laparoscopic hernia repair with mesh was performed; the patient achieved an uneventful postoperative recovery and reported no discomfort at the 1-month follow-up evaluation.

### 3.6. Study limitations

The main limitation of this report lies in its basis on a single case, which precludes the derivation of statistically significant conclusions and limits the generalizability of the observed clinical manifestations and treatment outcomes to a broader population. Furthermore, due to the short follow-up period (1 months), it was not possible to evaluate the long-term efficacy of the surgical intervention or the potential risk of late complications.

### 3.7. Summary

In summary, when patients present with abdominal trauma, surgeons should not focus solely on common visceral injuries but should also consider the possibility of rare conditions such as hepatic hernia. Although hepatic hernia may lack specific clinical manifestations, computed tomography is instrumental in its detection. Following definitive diagnosis, surgical intervention represents the definitive treatment. We have also summarized the relevant surgical approaches. Overall, the diagnosis and management of hepatic hernia pose considerable challenges. We present this case to contribute to the management of similar patients in the future.

## Author contributions

**Conceptualization:** Dezheng Liu, Lei Zhou.

**Data curation:** Dezheng Liu, Shukuan Yang.

**Formal analysis:** Shukuan Yang, Qinghai Guan.

**Funding acquisition:** Fan Zhang, Qinghai Guan.

**Investigation:** Zijian Zhao.

**Methodology:** Yangyang Cui.

**Project administration:** Yangyang Cui, Xuekai Zhao.

**Resources:** Fan Zhang.

**Supervision:** Fan Zhang, Qinghai Guan, Lei Zhou.

**Writing – original draft:** Dezheng Liu.

**Writing – review & editing:** Dezheng Liu, Lei Zhou.
